# Classical Philadelphia-negative myeloproliferative neoplasms: focus on mutations and JAK2 inhibitors

**DOI:** 10.1007/s12032-018-1187-3

**Published:** 2018-08-03

**Authors:** Grzegorz Helbig

**Affiliations:** Department of Hematology and Bone Marrow Transplantation, School of Medicine in Katowice, Medical University of Silesia, Dąbrowski street 25, 40-032 Katowice, Poland

**Keywords:** Driver mutations, Non-driver mutations, Myelofibrosis, Essential thrombocythemia, Polycythemia vera, JAK2 inhibitors, Ruxolitinib

## Abstract

Classical Philadelphia- negative myeloproliferative neoplasms (MPNs) encompass three main myeloid malignancies: polycythemia vera (PV), essential thrombocythemia (ET), and myelofibrosis (MF). Phenotype-driver mutations in Janus kinase 2 (JAK2), calreticulin (CALR), and myeloproliferative leukemia virus oncogene (MPL) genes are mutually exclusive and occur with a variable frequency. Driver mutations influence disease phenotype and prognosis. PV patients with *JAK2* exon 14 mutation do not differ in number of thrombotic events, risk of leukemic and fibrotic transformation, and overall survival to those with *JAK2* exon 12 mutation. Type 2-like CALR-mutated ET patients have lower risk of thrombosis if compared with those carrying *JAK2* or type 1-like *CALR* mutation. For ET, overall survival is comparable between patients with *JAK2* and either type 1-like and type 2-like *CALR* mutations. For MF, better OS is demonstrated for patients harboring a type 1-like *CALR* mutation than those with type 2-like *CALR* or *JAK2*. The discovery of driver mutations in MPNs has prompted the development of molecularly targeted therapy. Among JAK2 inhibitors, ruxolitinib (RUX) has been approved for (1) treatment of intermediate-2 and high-risk MF and (2) PV patients who are resistant to or intolerant to hydroxyurea. RUX reduces spleen size and alleviates disease symptoms in a proportion of MF patients. RUX in MF leads to prolonged survival and reduces risk of death. RUX controls hematocrit, reduces spleen size and alleviates symptoms in PV. Adverse events of RUX are moderate, however, its long-term use may be associated with opportunistic infections. Trials with other JAK2 inhibitors are ongoing.

## Driver mutations in MPNs

The classical Philadelphia (Ph)-negative myeloproliferative neoplasms (MPNs) encompass three main myeloid malignancies: polycythemia vera (PV), essential thrombocythemia (ET), and myelofibrosis (MF) [1]. Of note is, that phenotype-driver mutations in Janus kinase 2 (JAK2), calreticulin (CALR), and myeloproliferative leukemia virus oncogene (MPL) genes are mutually exclusive and occur with a variable frequency in patients with classical MPNs. Approximately 97% of PV patients carry a V617F mutation located in exon 14 of the *JAK2* gene. Those PV patients who are negative for V617F, may harbor mutation in exon 12. *JAK2*V617F mutation is also detected in 50–60% of ET and MF patients. About 20–25% of the patients with ET/MF harbor *CALR* mutation (exon 9) whereas mutation in exon 10 of the *MPL* gene is demonstrated in less than 10% of ET/MF cases. About 10% of either ET or MF patients are negative for all those three driver mutations [2].

In normal subjects, activation of JAK-STAT (the Janus kinase/signal transducers and activators of transcription) pathway is a consequence of ligand binding (e.g., erythropoietin) to cytokine receptors that leads to JAK proteins phosphorylation. The phosphorylated JAK proteins attract and phosphorylate STAT proteins which dimerize and enter the nucleus triggering expression of target genes causing cell growth [3]. The underlying mechanism by which driver mutations lead to myeloid proliferation results from cytokine-independent activation of JAK-STAT signaling pathway. All these three mutations have a gain-of-function effect on JAK-STAT signaling and are sufficient to induce myeloproliferative phenotype in mice models [4–7].

## Clinical correlates of driver and non-driver mutations

Driver mutations may have an impact on disease phenotype and prognosis. PV patients with *JAK2* exon 14 mutation do not differ in the number of thrombotic events, risk of leukemic and fibrotic transformation, and overall survival to those with *JAK2* exon 12 mutation [8]. Interestingly, a dozen different variants of *CALR* exon 9 mutations have been identified, but a 52-bp deletion (type 1) and a 5-bp insertion (type 2) are the most common. Type 2-like CALR-mutated ET patients are younger and have lower risk of thrombosis despite higher platelet count if compared with those carrying *JAK2* or type 1-like *CALR* mutation. The latter mutation is associated with higher risk of fibrotic transformation. JAK2-mutated MF patients are older and have lower platelet count when compared with CALR-mutated population. No difference in clinical features and risk of leukemic transformation (LT) is observed between ET and MF patients with type 1-like and type 2-like *CALR* mutations. ET patients carrying *JAK2* have highest risk of thrombosis. For ET, overall survival (OS) is comparable between patients with *JAK2* and either type 1-like and type 2-like *CALR* mutations. For MF, better OS is demonstrated for patients harboring a type 1-like *CALR* mutation than those with type 2-like *CALR* or *JAK2* [9]. MPL-mutated ET patients have lower hemoglobin levels and higher platelet count if compared with those without this mutation. The presence of *MPL* mutation is associated with a significant risk of vascular complications [10].

Recent studies have identified several non-driver mutations which have been shown to have a prognostic impact in patients with MPNs independent of well-known conventional risk factors. Of note is, that these additional mutations are not restricted to MPNs and can be detected in other myeloid malignancies [11]. The frequency and prognostic significance of other than *JAK2*/*CALR*/*MPL* mutations in PV/ET patients have been reported by Mayo Group. More than 50% of PV and ET patients were found to have at least 1 mutation other than well-described driver mutations and *TET2* and *ASXL1* were the most common. It was demonstrated that *ASXL1, SRSF2,* and *IDH2* for PV and *SH2B3, SF3B1, U2AF1, TP53, IDH2*, and *EZH2* for ET were associated with inferior survival, higher risk of leukemic, and fibrotic transformation. Of note is that the number of mutations does not carry prognostic significance [12]. For MF cohort, the presence of *ASXL1, SRSF2, IDH 1*/*2*, and *EZH2* mutations was found to have a negative impact on overall survival, but only *ASXL1* mutation remained significant independent of the well-validated dynamic international prognostic scoring system (DIPSS-plus) [13]. Unlike to what has been demonstrated in PV/ET, the number of these mutations negatively affected OS and leukemia-free survival [14]. A prognostic model based on the presence of high-risk molecular markers enables risk stratification for transplant-eligible MF patients [15]. The frequency and main clinical findings of commonly seen mutations in classical MPNs are presented in Table 1.

**Table 1:** Mutational frequency and main clinical findings of mutations in classic Ph(−) MPNs

	Mutational frequency %			Main clinical findings
	PV	ET	MF	
Driver mutations				
*JAK2* V617F	97	55	60	*JAK2*V617F: ↑age, ↑Hgb, ↑WBC, ↓PLT in ET and MF CALR: ↓age, ↑PLT, ↓Hgb, ↓WBC in ET CALR: ↓age, ↑PLT, ↓frequency of anemia and ↓leukocytosis in MF No difference in OS between *JAK2* V617F and *JAK2* exon 12 in PV *JAK2*V617F increases risk of thrombosis in ET *CALR* type-1: ↑risk of fibrotic transformation in ET *CALR* type-2: ↓risk of thrombosis in ET no difference in OS and LT between *CALR* and *JAK2* V617F in ET better OS in *CALR* type-1 vs *CALR* type-2, *JAK2* V617F and MPL in MF no difference in LT rates between CALR and *JAK2* V617F in MF *MPL* associated with small vessel disturbances in ET [9, 10, 13, 16]
*JAK2* exon 12	3	–	–	
*CALR*	–	25 Type-1 (57%) Type-2 (39%) Other (4%)	25 Type-1 (72%) Type-2 (16%) Other (12%)	
*MPL*	–	3	7	
Non-driver mutations				
*ASXL1*	12	11	22	*ASXL1, SRSF2, IDH2* associated with worse OS in PV *ASXL1, SRSF2, EZH2, IDH1*/*2* negatively correlated with OS and PFS in MF *IDH2* and *EZH2* associated with inferior OS in ET [3, 11–13]
*SRSF2*	3	2	8	
*EZH2*	-	3	5	
*IDH1*/*2*	2	3	5	

*ET* essential thrombocythemia, *Hgb* hemoglobin, *LT* leukemic transformation, *MF* myelofibrosis, *OS* overall survival, *PLT* platelets, *PV* polycythemia vera, *WBC* white blood cell

## Treatment of MPNs

Currently available therapies for PV and ET do not alter the natural history of diseases and are indicated to prevent thrombotic complications. Of note is, that cytoreductive treatment should be reserved for patients who have high-risk of thrombosis—e.g., for those who are > 60 years and/or have history of thrombosis. For MF patients, allogeneic stem cell transplantation (SCT) remains the only curative option, but due to high-risk of transplant-related mortality, it should be proposed after careful risk/benefit assessment. Unlike SCT, the other therapeutic options alleviate disease symptoms rather than modify disease course [16].

### *JAK2* inhibitors in MF

#### Ruxolitinib

The discovery of driver mutations in MPNs has prompted the development of molecularly targeted therapy. JAK2 inhibitors acting on JAK-STAT signaling cascade lead to increased cell apoptosis and reduction of cell proliferation [17]. Among JAK2 inhibitors, ruxolitinib (RUX)-dual JAK1/JAK2 inhibitor has been approved for the treatment of intermediate-2 and high-risk MF [18].

The primary end point of all MF trials was a reduction of 35% or more in spleen volume (SVR) from baseline at 24 or 48 weeks depending on study. The secondary end points included time to a SVR, progression-free survival (PFS), leukemia-free survival (LFS), and overall survival (OS). There have been COMFORT-1 and COMFORT-2 studies which evaluated the efficacy and safety of RUX in MF patients. In COMFORT-1 trial, treatment with RUX resulted in SVR ≥ 35% at 24 weeks in 42% of treated patients if compared with less than 1% in placebo group. In COMFORT-2 trial, SVR ≥ 35% at week 48 was noted for 28% of patients receiving RUX vs 0% in those who received best available therapy (BAT). Spleen reduction was maintained for more than 3 years in RUX patients either in COMFORT-1 and COMFORT-2 trials. The pooled-analysis of both studies has shown that the risk of death was reduced by 30% in RUX group if compared with the control. Moreover, patients who were originally randomized to RUX survived longer than those who crossed over from placebo/BAT to RUX [18–20]. The COMFORT trials have demonstrated that larger spleen size at baseline was associated with higher risk of death whereas spleen reduction on RUX resulted in better survival. Of note is, that patients with at least 25% spleen reduction on RUX had prolonged survival if compared with those without change in spleen volume [20, 21]. Moreover, it has been shown that RUX alleviated disease symptoms improving patient’s quality of life. An increased inflammatory responses have been demonstrated in patients with MPNs as a consequence of activation of a variety of cytokines. RUX was found to decrease serum cytokine levels reversing the constitutional symptoms thereby leading to clinical improvement [20–22]. These cytokines are produced by the mutated and non-mutated hematopoietic cells leading to progressive bone marrow fibrosis. Moreover, they seem to play a role in development of extramedullar hematopoiesis [23]. Interestingly, RUX treatment led to reduction of bone marrow fibrosis in 16% of MF patients in COMFORT-II trial [20]. MD Anderson group has reported on the results of bone marrow biopsies at baseline and at 24, 48, and 60 months of RUX. Improvement of fibrosis was demonstrated in 15%, 34%, and 36%, respectively. These results have proved that JAK inhibition modulates the cellular components of bone marrow microenvironment which are thought to be related to fibrosis [24]. Of note is, that only three cases with complete resolution of bone marrow fibrosis on RUX have been reported so far [25–27]. Long-term treatment with RUX is associated with opportunistic infections [28].

It was demonstrated that RUX altered the *JAK2* V617F allele burden in JAK2-positive patients with MF. Moreover, the decreases of allele burden correlated with spleen volume reduction and were inversely associated with disease duration. Only 11% of analyzed JAK2-positive MF patients achieved partial (PMR) or complete molecular response (CMR). Median time to PMR and CMR was 22 and 27 months, respectively. Interestingly, the spleen responses were also demonstrated in patients without allele burden reduction or in those who were JAK2- negative at baseline. Moreover, changes in allele burden did not correlate with improvement of blood parameters, clinical symptoms and bone marrow histopathology [29].

Treatment with RUX in MF can be associated with prolonged survival and reduced risk of death independent of mutation profile. Data from COMFORT-II trial have clearly demonstrated that RUX abrogated the negative impact of high-risk molecular markers. Of note is that these detrimental mutations did not decrease the probability of obtaining spleen reduction or improving clinical symptoms on RUX [30].

Anemia and thrombocytopenia are dose-dependent effects of RUX therapy. Grade 3/4 anemia and thrombocytopenia occurred in 45% and 13% of patients, respectively. One should keep in mind that RUX leads to drop in hemoglobin levels and platelet counts over the first 12 weeks of therapy with subsequent recovery. These RUX-related events require dose reductions or brief treatment interruptions as well as red blood cell or platelet transfusions [31]. Interestingly, the occurrence of RUX-induced anemia does not affect survival [32]. Non-hematologic side effects of RUX are mild and manageable. Of note is that two cases of secondary acute myeloid leukemia were reported in patients receiving RUX [31].

However, it should be noted, that data on RUX efficacy and safety for MF patients provided by Cochrane Systemic Review were not so evident as those quoted above. There was very low or low quality of evidence for the effect of RUX on OS and PFS when compared with placebo or BAT. Similarly, RUX did not decrease the risk of hematologic and non-hematologic toxicity when compared to comparators. The effect of RUX on reduction of spleen size remained uncertain. One should bear in mind that these conclusions were based on COMFORT-I/II industry sponsored trials and included a small number of patients [33].

#### Momelotinib

The safety and efficacy of momelotinib (MMB)- JAK1/2 inhibitor for intermediate and high-risk MF were evaluated in two randomized trials: SIMPLIFY-1 and SIMPLIFY-2. The former one compared MMB with RUX in patients who had not been treated with JAK2 inhibitors before. The primary study end point was similar to that of COMFORT trials. A proportion of patients who achieved ≥ 35% reduction in spleen size at 24 weeks was comparable between treated arms: 27% in MMB vs 29% in RUX. MMB was inferior when compared with RUX in terms of symptoms resolution. MMB treatment resulted in a significant decrease of transfusion requirement. Most common side effects of MMB included anemia and thrombocytopenia; 10% of treated patients developed grade ≤ 2 peripheral neuropathy [34]. The SIMPLIFY-2 study compared MMB with BAT in MF patients who had received previous RUX. MMB was not superior to BAT (including RUX) in decreasing spleen size (7% in MMB vs 6% in BAT group). Adverse events were similar between groups except neuropathy which occurred in 10% of MMB-treated patients and 0% in BAT population [35]. Recently, the Mayo Group have presented their results of a 7-year follow-up of MMB treatment in MF patients who were treated between 2009 and 2010 as a part of phase 1/2 study. A vast majority of patients (91%) discontinued treatment after median of 1.4 years, the remaining 9% are still on MMB with maximum duration of treatment exceeded 7 years. Common grade 3/4 adverse events of MMB treatment included thrombocytopenia and liver enzymes increase. Clinical improvements measured as spleen and anemia responses were demonstrated for 57% of MMB-treated patients. Majority of patients improved their quality of life. The presence of *ASXL1, SRSF2* and absence of *CALR* type-1 mutation was associated with unfavorable survival [36].

#### Pacritinib

Pacritinib (PAC) is a kinase inhibitor for *JAK2* and *FLT3*. Data from phase 1/2 trial study have shown that PAC induced spleen reduction with limited hematologic toxicity [37]. The PERSIST-1 study compared the efficacy of PAC (twice and once daily) vs BAT (excluding RUX) in high-risk MF patients. At 24 week SVR ≥ 35% was achieved by 19% patients in PAC group vs 5% in BAT (p = 0.0003) [38]. In the PERSIST-2 study, patients with myelofibrosis and platelet count ≤ 100 × 10^9^/L were randomized to PAC or BAT (including prior RUX). PAC was found to be more effective than BAT in SVR ≥ 35% (18% vs 3%) and led to greater than 50% reduction in total symptom score (25% vs 14%). Of note is, that Food and Drug Administration (FDA) in 2016 placed a full clinical hold on PAC studies following fatal complications including cerebral bleeding and cardiac failures. In January 2017 FDA has lifted its hold on PAC and a new dose-finding study PAC203 was initiated [18, 39]. Patients who benefited from PAC in PERSIST studies were allowed to continue the treatment on compassionate-use basis. Re-initiation of PAC resulted in mild improvement in SVR with low rate of hematologic toxicity. It seems reasonable to offer PAC for patients with MF and pancytopenia, especially thrombocytopenia [40].

#### Fedratinib

Fedratinib (FED) is a JAK2 inhibitor which efficacy was first evaluated in the JAKARTA-1 study. FED at two doses (400 mg and 500 mg) was compared with placebo in 289 patients with intermediate-2 or high-risk MF. Spleen response was achieved in 36% of FED-treated patients at 400 mg, 40% at 500 mg and 1% in the placebo group. Four patients receiving FED at 500 mg daily developed Wernicke encephalopathy (WE) [41]. The JAKARTA-2 study included patients with MF who were resistant to or intolerant to RUX. Spleen response was noted in 53% of patients who were RUX resistant and 63% of patients who were RUX intolerant. However, it should be mentioned that 7% of patients died during the study as a consequence of encephalopathy. It was the reason for study discontinuation [42]. Recently, the analysis of 9 FED trials with 670 patients has revealed that the prevalence of encephalopathy in this population is less than 1%. Based on this data, FDA decided to lift the clinical hold [43].

### *JAK2* inhibitors in PV/ET

#### Ruxolitinib in PV

RUX has been approved for PV patients who are resistant to or intolerant to hydroxyurea based on a phase 3 study (RESPONSE) comparing RUX with BAT. The primary end point was both hematocrit control and ≥ 35% SVR reduction and at week 32. Hematocrit control was observed in 60% of patients in RUX group when compared to 20% of those in BAT arm. At least a 35% SVR achieved 38% and 1% patients, respectively. There was a significant difference in complete hematologic remission (CHR) rate: 24% in RUX vs 9% in BAT. Moreover, the reduction in total symptom score was greater in RUX patients. Side effects were manageable and similar to that presented in COMFORT trials [44]. The RESPONSE trial has also focused on long-term effect of RUX treatment on *JAK2*V617F allele burden. JAK2-positive PV patients were randomized to RUX or BAT with cross-over to RUX at week 32. It was demonstrated that mean decrease of *JAK2* allele burden from baseline ranged from − 12 to − 40% (RUX arm) and − 6 to − 18% (RUX cross-over). CMR and PMR were demonstrated in 3 and 54 patients, respectively. However, one should bear in mind that the reduction in allele burden did not correlate with clinical outcome. These findings were consistent with those seen for MF [45]. Recently, a 4-year follow-up of RESPONSE study has been presented. The median duration of CHR was not reached and 54% of patients remained in CHR. The estimated 5-year OS rates were comparable between arms. Hematologic toxicity improved with RUX continuation as well as non-hematologic adverse events. Infection was the most common side effect of RUX. Rates of thrombotic events were low in both arms [46].

#### Ruxolitinib in ET

The MAJIC-ET study compared the efficacy of RUX vs BAT for ET intolerant or resistant to HU. Fifty-eight patients were randomized to RUX, however, there was no evidence of superiority of RUX when compared to BAT at 12 months [47]. In contrast, MD Anderson study has demonstrated that RUX treatment was associated with the improvement in ET-related symptoms as well as better and durable platelet, leukocyte, and hemoglobin control. The reduction of *JAK2-*allele burden was also noted [48].

#### Momelotinib in PV/ET

A phase 2 trial evaluated MMB in patients with PV and ET, however, it was prematurely discontinued due to limited efficacy [49]. The results of studies with JAK2 inhibitors in classical MPNs are shown in Table 2.

**Table 2:** JAK2 inhibitors in clinical trials

Compound	Comparator	Trial	Indication	Primary end point
RUX	PBO	COMFORT-1 [19]	MF	SVR ≥ 35% at 24 week: 42% (RUX) vs 1% (PBO)
RUX	BAT	COMFORT-2 [20]	MF	SVR ≥ 35% at 48 week: 28% (RUX) vs 0% (BAT)
MMB	RUX	SIMPLIFY-1 [34]	MF	SVR ≥ 35% at 24 week: 27% (MMB) vs 29% (RUX)
MMB	BAT (incl. RUX)	SIMPLIFY-2 [35]	MF	SVR ≥ 35% at 24 week: 7% (MMB) vs 6% (BAT)
MMB	BAT	RESPONSE [44]	PV	Hematocrit control at week 32: 60% (RUX) vs 20% (BAT) SVR ≥ 35% at 32 week: 38% (RUX) vs 1% (BAT)
PAC	BAT	PERSIST-1 [38]	MF	SVR ≥ 35% at 24 week: 19% (PAC) vs 5% (BAT)
PAC	BAT (incl. RUX)	PERSIST-2 [39]	MF	SVR ≥ 35% at 24 week: 18% (PAC) vs 3% (BAT)
FED	PBO	JAKARTA-1 [41]	MF	SVR ≥ 35% at 24 week: 36% (FED400) vs 40% (FED500) vs 1% (PBO)
FED	Single arm (RUX resistant/intolerant)	JAKARTA-2 [42]	MF	SVR ≥ 35% at 24 week: 55%
RUX	BAT	MAJIC-ET [47]	ET	CRR at 12 months: 47% (RUX) vs 44% (BAT)

*CRR* complete response rate, *BAT* best available therapy, *FED* fedratinib, *MMB* momelotinib, *PBO* placebo, *PAC* pacritinib, *RUX* ruxolitinib, *SVR* spleen volume response

### *JAK2* inhibitors before stem cell transplantation

Patients with intermediate-2 and high-risk MF remain candidates both to RUX and SCT. Currently, the majority of transplant-eligible patients are treated with RUX before SCT in daily clinical practice. Several studies reported on the outcome of SCT for MF with prior exposure to RUX [50, 51]. Among 66 patients who discontinued RUX before SCT, 2 patients required SCT delay due to serious adverse events (AEs) It was concluded that RUX should be stopped just before conditioning commencement. AEs were more common in those who discontinued treatment ≥ 6 days before conditioning. Treatment with RUX did not negatively influence outcome after SCT [50]. There is a little experience with RUX after SCT. It was demonstrated that RUX may prevent the occurrence of acute graft vs host disease, however, 40% of transplanted patients developed cytomegalovirus reactivation [52].

## Conclusions

Discoveries of driver and non-driver mutations in MPNs allow to simplify diagnostic algorithm as well as better define disease prognosis. They may also serve as drug targets. Current experience with JAK2 inhibitors is practically restricted to patients with intermediate and high-risk MF. These compounds alleviate disease symptoms and reduce spleen size in a proportion of patients with MF. Their impact on overall survival is debatable. RUX reduces hematocrit and spleen volume in PV patients, but was found ineffective in ET. Long-term use of RUX has been associated with opportunistic infections. SCT still remains only curative treatment for MF. Peritransplant role of RUX has to be elucidated.
